# Physical Properties of Starch/Powdered Activated Carbon Composite Films

**DOI:** 10.3390/polym13244406

**Published:** 2021-12-15

**Authors:** Anita Kwaśniewska, Michał Świetlicki, Adam Prószyński, Grzegorz Gładyszewski

**Affiliations:** Department of Applied Physics, Lublin University of Technology, 20-618 Lublin, Poland; m.swietlicki@pollub.pl (M.Ś.); a.proszynski@pollub.pl (A.P.); g.gladyszewski@pollub.pl (G.G.)

**Keywords:** starch composite film, activated carbon powder, mechanical properties, AFM, X-ray diffraction, barrier properties

## Abstract

In the present study, starch/powdered activated carbon composite films were prepared by incorporating various amounts of powdered activated carbon (PAC)—1–5, 10, and 15 %—into a starch matrix, using the solvent casting method. The effect of PAC addition on the biopolymer film was investigated. The mechanical properties were examined by ultra-nanoindentation, nanoscratch, and micro-tensile tests. Since the mechanical properties of biopolymer films are correlated with their structure, the effect of PAC addition was tested using X-ray diffraction. The surface parameters morphology and wettability were analyzed by atomic force microscopy (AFM) and contact angle measurements. The barrier properties were examined by determining water vapor permeability and the water solubility index. The obtained results did not show a monotonic dependence of the mechanical parameters on PAC content, with the exception of the maximum strain, which decreased as the amount of the additive increased. The visible effect of PAC addition was manifested in changes in the adhesive force value and in water vapor permeability (WVP). The barrier properties decreased with the increase of the filler content.

## 1. Introduction

Due to its biodegradability, abundancy, renewability, and economic aspects, starch is recognized as an environmentally friendly polymer. Moreover, as a result of its thermoplastic properties, it can be used as a comprehensive industry material. Films based on starch biopolymers have already been extensively investigated in order to develop new materials for sustainable development. Nevertheless, starch-based materials are hydrophilic and have poor vapor barrier and mechanical properties [1]. Therefore, these drawbacks have to be overcome to improve the performance of starch-based biomaterials.

Various experimental tests can be carried out to examine the properties of starch composites to suit specific applications, including food contact packaging materials, components used to protect industrial products [2,3], films applied in agriculture, membranes, bedding materials, and sorption mats [4,5]. Several attempts were made to improve starch-based material features by adding functional fillers, leading to improvements in physical, mechanical, and barrier properties. Nano- and micro-additives, both organic and inorganic, are used to produce starch-based biocomposites [2]. However, according to numerous studies, nanoadditives match the base polymer matrix best [6]. Regardless of the application, biodegradable materials should be enriched with non-toxic, readily available, inexpensive, and easy-to-process additives. The most popular nanoadditives which meet these requirements are aluminosilicates, especially, layered silicates such as montmorillonite [6], kaolin [7], and talc [8]. Furthermore, to change the properties of starch films, TiO_2_ and Ag nanoparticles can also be added [9,10].

Activated carbon seems to be one of the promising additives for starch-based biomaterials. It is a non-toxic, relatively inexpensive compound and, as reports show, has many additional physico- and electro-chemical properties. Powdered activated carbon has a microcrystalline structure, which has been processed to develop internal porosity. Increased porosity results in an increase in the absorbing surface [11,12]. It is a material with high absorption capacity for various chemical compounds [13,14]. It allows PAC to be used as a biosensor [15], widely applied in water [16,17] and air purification processes as well as in advanced oxidation procedures. PAC is also known for its odor-absorbing properties. It is used for the adsorption of wound malodor in absorbent dressing [18,19].

The present research aimed to investigate the effect of the addition of activated carbon powder on the mechanical, barrier, and surface properties of a composite based on a natural polysaccharide polymer. As a result, the colorless, tasteless, and odorless starch polymer was modified bay adding different concentrations of PAC equal to 1%, 2%, 3%, 4%, 5%, 10%, and 15%. The effect of PAC addition on the structure, surface parameters, and wettability of the starch-based films was investigated using X-ray diffraction, atomic force microscopy (AFM), and contact angle measurements. Mechanical parameters were determined by micro-tensile, nanoindentation, and scratch tests, whereas barrier properties were obtained by calculating water vapor permeability and the water solubility index.

## 2. Materials and Methods

### 2.1. Material

The raw material used to prepare the biopolymer films was potato starch produced by Melvit S.A. (Warsaw, Poland). The native starch was a raw product unmodified in any chemical, physical, or enzymatic way. Distilled water was used as a solvent in which the polymer solution was prepared. Glycerol 99.5% produced by Avant Performance Materials was used as the plasticizer. Powdered activated carbon (PAC) CWZ-22 (StanLab, Lublin, Poland), 100 mesh, was used as a nanofiller.

#### Film Preparation

The preparation of the biopolymer films was achieved with the casting method [20]. To obtain a uniform suspension, carbon powder added into 15 mL of distilled water, was treated with an ultrasonic homogenizer for 180 s at 25 °C [7]. The obtained suspension was poured into a prepared 105 mL aqueous solution of starch and plasticizer (20% w/w) and mixed with a magnetic stirrer rotating at 150 rpm for 30 min, heating up to 80 °C. The final solution was poured into molds and kept in a climatic chamber until the solvent evaporated. Sample drying was carried out at 23 °C at relative humidity of 45% (RH) for 4 days. Afterwards, the films were stored in a desiccator and maintained at 50% RH. Finally, samples containing 0–5%, 10%, and 15% of PAC with respect to dry starch were produced. The obtained films were respectively coded as follows: C0–C5, C10, and C15.

The thickness of the obtained films was determined using an electronic micrometer (Limit, Alingsås, Sweden). The measurements were performed at 10 randomly selected points of each sample, and the mean with ±SD is presented.

### 2.2. X-ray Diffraction (XRD)

The powder X-ray diffraction patterns of all samples were recorded using a high-resolution X-ray diffractometer (Empyrean, Panalytical BV, Almelo, The Netherlands) in Bragg–Brentano geometry, using CuK_α_ (l = 1.5418 Å) radiation of tube operated with a generator voltage of 40 kV and a current of 30 mA. The patterns of the samples were recorded in the 2θ range of 10–70°, with a step size of 0.01° and a count time of 8 s per data point. The radiation was detected with a proportional detector. The source divergence and detector slit were ½, and Soller’s slits were applied. X’Pert HighScore Plus software was used for the phase analysis. Grapher 18 (Golden Software LCC, Golden, CO, USA) was used to plot the diffraction profiles.

### 2.3. Mechanical Properties

#### 2.3.1. Tensile Test

The Young’s modulus, tensile strength, and maximum strain were determined from the uniaxial tensile tests. The measurements were performed with a Deben Microtest tester (Deben Ltd., Suffolk, UK) equipped with a 200 N maximum load cell. The samples were rectangular, with a width of about 3.5 mm (the exact value was measured with an accuracy of 0.01 mm for each sample) and a length of 20 mm. The thickness of the samples ranged from 0.060 mm to 0.110 mm and was measured before each test with an accuracy of 0.001 mm. The initial distance between the grips was always 11 mm, and the samples were elongated at a rate of 0.5 mm/min. Five independent tensile tests were performed for each sample.

#### 2.3.2. Nanoindentation Test

The tests to determine the nanomechanical properties were performed using an Ultra Nano-Hardness Tester with a Berkovich indenter (Anton Paar GmbH, Graz, Austria). The obtained values of hardness (H_UNHT_) and modulus of elasticity (E_UNHT_) were based on the Oliver–Pharr model and were calculated directly from the indenter force and the displacement curves [21]. The hardness is the ratio of the applied force to the projected indentation impression area determined from the contact depth, whereas the modulus of elasticity is the relationship between stress and strain when deformation is elastic [22]. The nanoindentations were carried out with a linear load change at a rate of 10 mN/min with a 10 s pause between increasing and decreasing the load at a depth of 5000 nm. The minimum contact force was 0.1 mN. Each measurement consisted of four indentations, carried out in places separated by a stretch 10 times the size of the imprint to avoid the influence of the indentations on each other. The indentation depth was 10% of the total thickness of the thinnest C0 sample to avoid the impact of the substrate. The hardness (H_UNHT_), modulus of elasticity (E_UNHT_) values, and the corresponding average values were calculated for each test. The measurements were carried out at 23 °C and 50% RH.

#### 2.3.3. Nanoscratch Test

The nanoscratch test was performed using a Nano Scratch Tester by CSM Instruments, with a conical indenter (Anton Paar GmbH, Graz, Austria). The test was performed with force increasing from 0 to 50 mN, with the scratch length of 1.2 mm at a constant speed of 0.12 mm/min. The maximum force was selected so that the sample would not be delaminated from the substrate. Four tests consisted of three scans scratches for each sample. The first scan (Prescan) was carried out to obtain the original surface profile. The second scan was the actual scratch process (Penetration depth), and the third scan (post-scan) showed the final surface profile after the scratch (Residual depth). The results obtained during the scratch test were used to calculate the elastic recovery (ER**_NST_**) of the film after deformation, in accordance to Equation (1) in Ref [23]. In addition, the roughness values R_a_ and R_ms_ were determined using each sample’s profiles measured during the Prescan.

### 2.4. Surface Properties

#### 2.4.1. Atomic Force Microscopy

A MultiMode 8 with a NanoScope V controller equipped with a J-type scanner (Bruker, Billerica, MA, USA) was used to conduct the AFM measurements in the PeakForce QNM mode. The measurements were carried out under ambient conditions at 23 °C and 50% RH to characterize the film’s morphology and mechanical properties at the nanometric scale.

A Vtespa 300 (Bruker, Billerica, MA, USA) probe, characterized by a nominal spring constant k = 42 N/m, a tip radius R = 5 nm, a length of 140 µm, and a width of 38 µm, was used for all measurements. The deflection sensitivity was measured with a sapphire surface. The spring constant k was calibrated using the thermal tuning method, and R was evaluated using the absolute method on Bruker’s test samples. All measurements were performed for three noncontiguous areas of 10 μm × 10 μm in each sample. A digital resolution of 512 px × 512 px and a scanning rate of 0.5 Hz were used, and the z-range was set at 300 nm. The Young’s modulus was determined by fitting a linearized Derjaguin–Muller–Toporov (DMT) model to the contact part of the retraction curve [24,25]. Additionally, the adhesion force, identified by the attraction between the AFM tip and the sample surface, was determined.

#### 2.4.2. Wettability

Contact angle measurements based on a sessile drop method were applied to determine the wettability of the surfaces of the films [7]. An Attension Theta Lite optical goniometer (Biolin Scientific, Espoo, Finland) was used to determine the contact angle from the droplet geometry at 23 °C and 50% RH. A drop of deionized water (3 μL) was placed on each film, each time on a new surface. The image was captured 5 s after the drop was deposited on the surface. The measurement series consisted of 10 deposited drops. The mean value read from both sides of the drop was taken as the measured value of the contact angle.

### 2.5. Barrier Properties

#### 2.5.1. Water Vapor Permeability (WVP)

**Figure 1:**
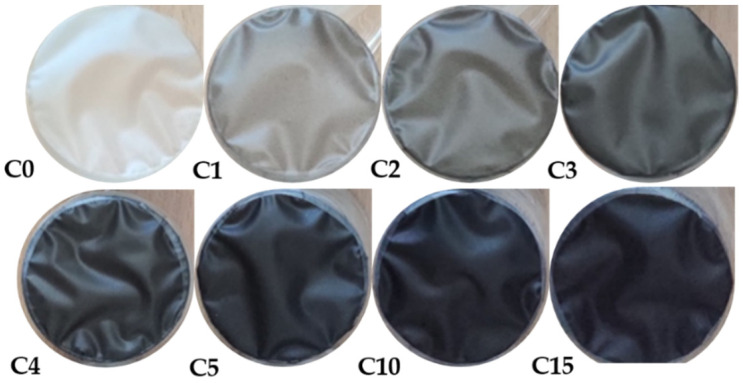
Image of starch composite film-covered vessels for the WVP test.

Water vapor permeability (WVP) was measured using the gravimetric method, based on measuring the loss of mass from a film-covered vessel over time under strictly defined conditions (Figure 1). The containers were filled with distilled water and sealed with Parafilm^®^M film. The surface area of the film, which the vapor could penetrate, was equal to A = 9.616 × 10^−4^ m^2^. The samples after preparation were weighed and kept in a climate chamber at 23 °C and 40% relative humidity. The test was conducted for 7 days, with daily weight measurements. The WVP was calculated using Equation (1) [26]. Each result was the average of two measurements.

*WVP* = (Δ*m·e*)/(Δ*t·A·*Δ*p*), (1)

where: Δ*m*/Δ*t* is the weight of moisture loss per unit of time [gs^−1^] determined from the slope obtained from the regression analysis of weight loss data versus time; *A* is the film area exposed to the vapor penetrate (9.616 × 10^−4^ m^2^); *e* is the film thickness [m]; Δ*p* is the water vapor pressure difference between the two sides of the film [Pa].

#### 2.5.2. Water Solubility Index (WSI)

The films were cut into 20 mm × 20 mm pieces, subsequently dried in an oven at 50 °C for 24 h to determine their dry mass. Previously dried and weighted samples were put into a beaker filled with 50 mL of H_2_O_dest_ at 24 °C, left there for 24 h, and stirred at 50 rpm. Then, the insoluble films were dried for 24 h at 50 °C again and weighed. The WSI index was calculated according to Equation (2) [9]:

*WSI =* ((*m*_1_ − *m*_2_)/*m*_1_) 100%, (2)

where: *m*_1_ is the initial dry film weight [g], and *m*_2_ is the final dry film weight [g]. For every film, measurements were conducted in triplicate.

### 2.6. Statistical Analysis

The obtained data were analyzed using Statistica 13.1 (TIBCO Software Inc., Palo Alto, CA, USA) application. One-way ANOVA and post-hoc tests (Tukey) were used to assess the differences in the mean values of the examined parameters. Data are given as means with the corresponding standard deviation (±SD).

## 3. Results and Discussion

**Figure 2:**
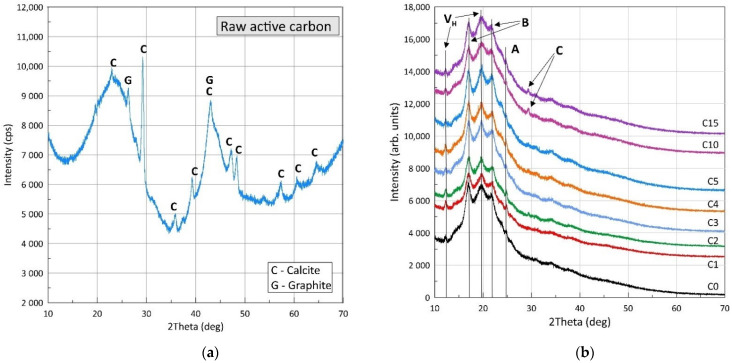
(**a**) XRD pattern of a raw powdered activated carbon; (**b**) XRD pattern of (**a**) thermoplastic starch samples containing 0% (C0), 1% (C1), 2% (C2), 3% (C3), 4% (C4), 5% (C5), 10% (C10), and 15% (C15) powdered activated carbon.

The results of the X-ray diffraction (XRD) measurement of raw active carbon powder and thermoplastics starch (TPS) with different percentages of raw PAC are shown in Figure 2a,b. In the X-ray diffraction patterns of raw active carbon powder (Figure 2a), one can distinguish two very wide diffraction peaks at about 2θ = 25° and 44°, corresponding to the (002) and (001) crystal planes of carbon, and a few peaks with relatively narrow half-widths. The presence of wide peaks indicates a significant content of the amorphous phase in the structure of raw active carbon. However, the presence of narrow peaks indicates the content of the crystalline phase in the tested powder.

The crystalline phase in PAC usually consists of graphite and various impurities depending on the source of the activated carbon. Phase analysis showed that, apart from the graphite peaks (G) occurring at around 2θ = 26.4°, the more intense diffraction peaks (C) were derived from calcite (see Figure 2a) [27]. It is worth noting that the height of the diffraction peaks was proportional not only to the phase content, but also to the electronic density, which is higher for heavy elements compared to carbon. This explains the height intensity of the calcite peaks compared to the graphite ones.

All XRD patterns generally showed the same shape and crystalline diffraction peaks at the same positions. Additionally, one can observe one wide peak (diffuse pattern) around 2θ = 20°. The presence of a wide amorphous peak and narrow crystalline peaks indicate a mixed amorphous and crystalline structure of the film. The occurrence of peaks in characteristic positions at 14.9° and 22° indicated that in TPS films there is mainly a B-type crystalline structure [28]. On the other hand, the occurrence of peaks at around 24° indicates the presence of the A-type crystal structure [29]. Peaks at positions 2θ = 12.5° and 19.6° indicate the presence of V_h_-type crystal structures resulting from the manufacturing process [30]. The peak marked C (see Figure 2b) is derived from calcite. The structure of type B is hexagonal, with the space group P6_1,_ and is derived from amylose, and the structure of type A is orthorhombic, with the space group P2_1_2_1_2_1_ which originated from cereal starch.

The X-ray results show that the powdered activated carbon did not intercalate with the starch biopolymer matrix, causing low interfacial adhesion between polymer matrix and filler.

When performing the uniaxial tensile tests, the relationship between sample strain and tensile force was recorded. Tensile strength σ_max_, Young’s elastic modulus E, and maximum strain ε_max_ were accessible directly from these measurements. The determined parameters and results of their statistical analyses are reported in Table 1. The effect of powdered activated carbon content on the mechanical parameters of the films, as shown in Table 1, is visible. When the tensile strength σ_max_ was between 1.86 to 3.42 MPa without any clear additive dependence, the maximum strain dropped nearly monotonically from 0.53, for the C0 sample, to 0.16, for the C15 sample. For low powdered activated carbon doses, the Young’s modulus ranged from 17.95 MPa to 45.72 MPa. However, for samples C10 and C15, it reached the values of 79.65 MPa and 55.08 MPa, respectively.

The presented results are in line with those achieved by Cyras P. et al. [31], who examined starch/montmorillonite (MMT) nanocomposite films. For nanocomposite with 2% and 3% of MMT, the tensile strength (σ_max_) value was lower than that determined for the basic starch film. Further, the increase of MMT (5%), caused the growth of σ_max_. In comparison, the highest ε_max_ was achieved by the film without MMT addition. Subsequently, ε_max_ decreased for the 5 and 2% films and reached the smallest value in the presence of 3% MMT. Similar results were found by Zao L. et al. [32], who observed a nonlinear relationship between increasing activated carbon amounts and the tensile strength (σ_max_) value for chitosan/activated carbon gel films. The parameter σ_max_ rose for 1% PAC and afterwards decreased (3% PAC) and increased again (5, 8, and 10% PAC).

The mechanical properties of starch/PAC films stemming from the filler did not intercalate in the biopolymer matrix. The results of the diffraction tests shown in Figure 2b were confirmed. Prakash M.O. et al. [33] noticed that PAC tends to agglomerate. The aggregation is a multi-scale problem related to the morphology, mass, and chemical properties of the nanoparticles [34]. However, studying the aggregation process was outside the scope of this research. The main goal of this work was the determination of the effect of PAC addition on the physical properties of polymer films. The obtained results showed no monotonic relationship between the physical properties and the amount of the additive. In further research, we intend to analyze the material’s internal structure, focusing, inter alia, on the agglomeration of the additive particles, which decrease the effective contact area between PAC and the biopolymer matrix and act as internal stress concentrators. The aforementioned effect impacts the composite’s mechanical properties, causing changes in the values of σ_max_ and ε_max_.

The hardness (H_UNHT_) and modulus of elasticity (E_UNHT_) values obtained from the nanoindentation tests are shown in Table 1. It was observed that 1–3% of PAC in the composite reduced H_UNHT_ compared to that 5.75 MPa for C0 film. In addition, a further increase in filler loading caused an increase in hardness up to 16.51 MPa for the C15 film. Similarly, the elasticity value E_UNHT_ reached a minimum in the presence of 1–3% PAC, corresponding to 19.50, 32.25, and 30.25 MPa, respectively. The highest E_UNHT_ value was measured for the C15 film. The values of the Young’s modulus E obtained from the tensile and nanoindentation tests, shown in Table 1, varied considerably. However, McKee C.T. et al. [35] reported that the mechanical parameters determined by the nanoindentation and tensile tests vary significantly because nanoindentations test the material’s surface, whereas the tensile test engages the material as a whole.

The increase in PAC content in the composite did not significantly affect the value of the elastic recovery ER**_NST_**, except for the C10 film. The highest value (0.89) was measured for the C0 film, and the lowest, ER**_NST_** = 0.55, for the C10 film.

**Figure 3:**
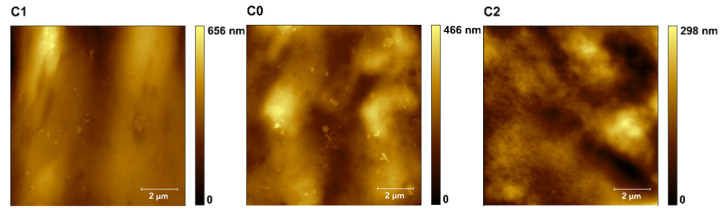

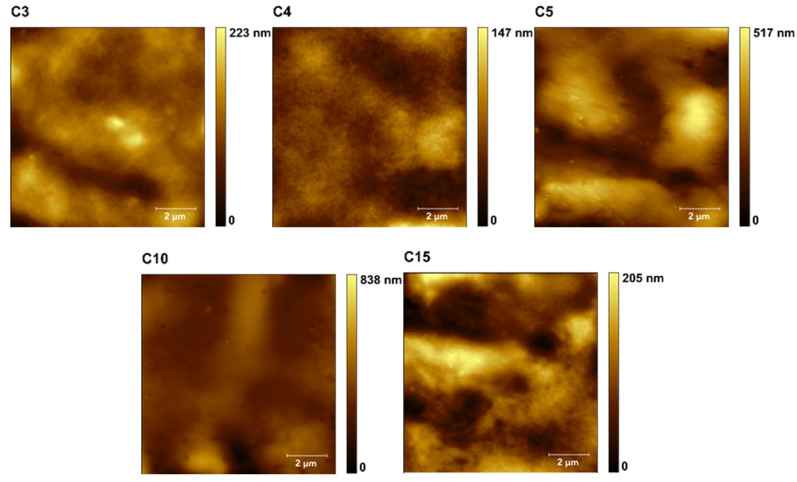
Representative maps of the topography (10 × 10 μm) of samples C0–C5, C10, and C15.

**Figure 4:**
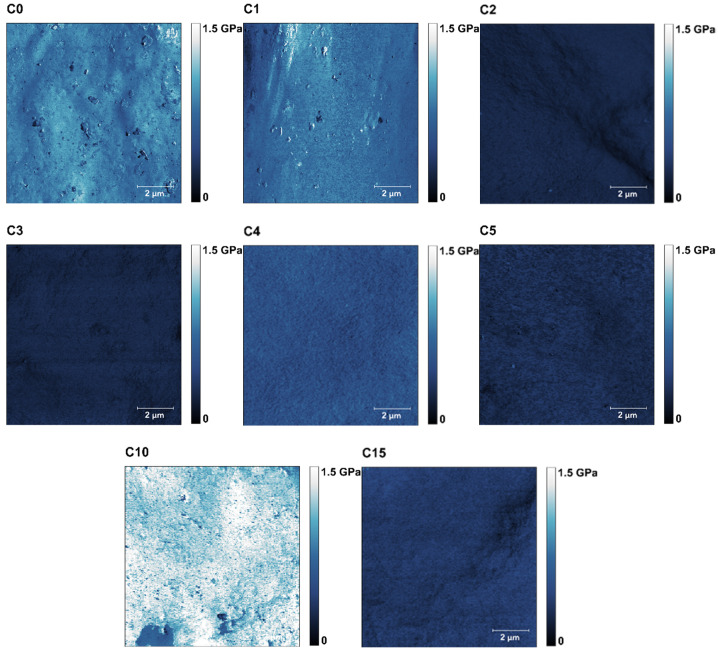
Representative maps of the DMT moduli forces (10 × 10 μm) for the samples C0–C5, C10, and C15.

**Figure 5:**
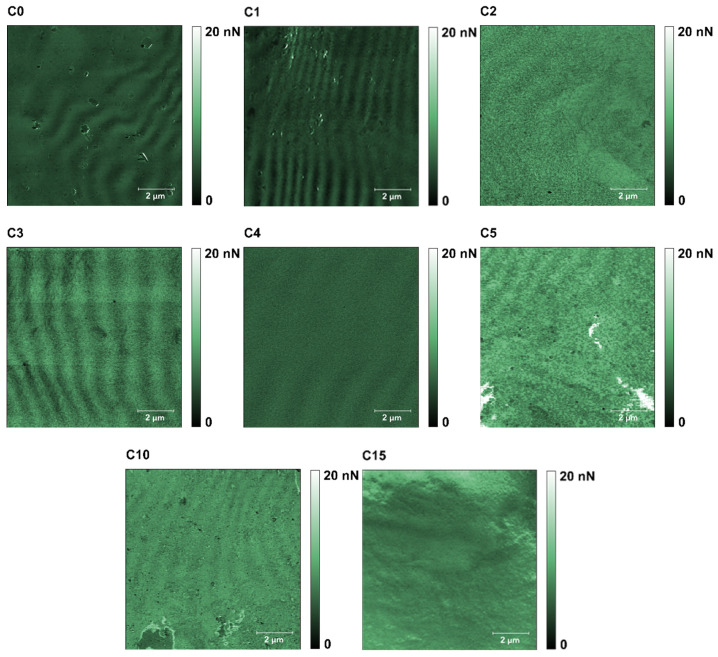
Representative maps of the adhesion forces (10 × 10 μm) for the samples C0–C5, C10, and C15.

The results obtained from the AFM measurements are presented in the form of maps of height (Figure 3), DMT modulus (Figure 4), and adhesion force (Figure 5), obtained by Gwyddion 2.59 software application. The mean values of the DMT modulus and adhesion force were based on every single point of the 512 × 512 scans, therefore in all cases, the number of counts was equal to 262,144. Data from three noncontiguous areas for each of the tested samples were used for the analysis.

Representative 2D maps of surface topography in the height domain are presented in Figure 2. The images were corrected to eliminate irregularities associated with the shape of the samples (3rd order plane fit). The surface morphology did not change with the increase in the nano-additive content in the biopolymer matrix, as shown in Figure 3, imaged by Gwyddion 2.59 software. No significant changes were observed in surface topography (Figure 3), as the additive content increased. Changes in the DMT modulus were similar to changes noted with the uniaxial tensile tests.

For samples C0 and C1, the DMT modulus values (Figure 4) were very similar and amounted to 686 and 637 MPa, respectively. However, an apparent decrease in the modulus was observed for samples C2, C3, and C5. On the other hand, for samples C4, C10, and C15, a significant increase in the modulus was observed (1052, 1400, and 772 MPa, respectively).

A significant effect of the additive on the adhesion force (Figure 5) was observed only for the samples with additive content above 1%. For C0 and C1, the force values were similar and amounted to 4.05 and 3.30 nN. The highest adhesion force values were obtained for samples C2, C3, and C5 (10.75, 13.70, and 10.72 nN, respectively). The measured forces were slightly lower for the C4, C10, and C15 samples and amounted to 7.51, 8.79, and 6.79 nN, respectively.

Surface hydrophobicity was determined by the water contact angle on the film surface. The results of the tests are presented in Table 2. According to Bhushan B. and Jung Y.C. [36], if a liquid wets a surface (wetting liquid or hydrophilic surface), the contact angle value is in the range 0° ≤ θ ≤ 90°, if this does not occur (non-wetting liquid or hydrophobic surface), the value of the contact angle is 90° < θ ≤ 180°. Therefore, small water contact angles (<90°) represent good wettability and, thus, a hydrophilic surface. On the other hand, angles >90° characterize a hydrophobic surface. In addition, wettability is determined by the surface’s chemistry and morphology (structures). Roughness changes the contact angle in accordance with the Wenzel model or Cassie–Baxter model [36]. Due to its chemical structure, starch has hygroscopic properties. Therefore, it contributes to the hydrophilic features of a starch film surface, which is wetted by water. Surface wettability measurements confirmed that for the C0 film, for which the contact angle was equal to 70°. At the same time, activated carbon has hydrophobic properties. PAC addition to the films increased the contact angle, with a relatively small change in roughness from 2 µm to 2.58 µm. In the presence of 1% PAC, the contact angle increased to 86°, and the surface was still hydrophilic according to the classification, while the surface of the C2–C5 films became hydrophobic, with the observed contact angle within the range of 100°−102°. A further increase of PAC addition to 10% and 15% caused the surface roughness to almost double for C10 (Rms = 3.64 µm) and increase even more for C15, for which Rms = 5.44 µm. The significant increase in roughness caused a rise in the surface wettability; hence, the films became hydrophilic. A similar nonlinear contact angle dependence on the percentage content of the filler was obtained by Oleyaei et al. They examined starch-based films enriched with hydrophobic TiO_2_ nanoparticles, for which an increase in additive also increased the nanocomposite surface roughness [9].

The effect of powdered activated carbon addition on water vapor permeability of the biopolymer films is shown in Table 2. For all composite films, it can be observed that the barrier property decreased when increasing the filler content. The lowest measured WVP value was obtained for C0, 2.60 [10^−10^ g/(m·s·Pa)], and the highest one for the composite film C15, 4.13 [10^−10^ g/(m·s·Pa)]. The WVP index strictly depends on the quantity of –OH groups in the polymer because water vapor transfers through the hydrophilic part of the matrix [37]. Since the addition of carbon does not change the structure of the matrix, there was no intercalation, which was confirmed by the X-ray diffraction results. The amount of hydrogen bonds available for water vapor was not reduced. In addition, the carbon powder tends to aggregate at higher concentrations, as observed by Prakash, M.O. et al. [33]. The increase of WVP could have been due to the agglomeration of PAC in the starch matrix. The aggregation caused by a high filler content probably created a macro-phase separation through which vapor diffusion increased.

The reduced WVP is of great importance when the packaged product has a long shelf life; in contrast, vapor-permeable films do not condense excess moisture in the package, so they can replace the absorption pads in short-lifespan goods containers.

The water solubility WSI of biocomposite films containing various concentrations of PAC are shown in Table 2. The solubility increased with the increase of the additive, reaching the highest value for C4 and C5, while it decreased in the presence of the highest amounts of additive, i.e., 10% and 15%. This may be due to the water-insoluble hydrophobic PAC taking up more space in the specimen than the more soluble starch matrix. In addition, the C10 and C15 films were thicker. Therefore, PAC particles can form an insoluble barrier protecting the biopolymers.

Further research may focus on the determination of the adsorption efficiency of the obtained films. Furthermore, due to the characteristic structure of activated carbon, it can be assumed that the biocomposite obtained in this way may have additional protective properties as innovative packaging or as a biodegradable sorption mat, e.g., for heavy metal ions.

## 4. Conclusions

This paper presents the results of research on a new starch biocomposite. In order to change the physical properties of the biopolymer, the addition of powdered activated carbon was applied in the proportions of 1–5%, 10%, and 15% in relation to the dry starch weight. As a result, the obtained films significantly changed their color, from transparent for the C0 sample to completely black and opaque for the C15 one.

The starch-based biopolymer enriched with powdered activated carbon was examined by ultra-nanoindentation, nanoscratch, and micro-tensile tests. Since the mechanical properties of biocomposite films are known to be correlated with their structures, the effects of PAC addition were investigated using X-ray diffraction. The surface morphology and wettability were investigated by atomic force microscope (AFM) and contact angle measurements. Barrier properties were determined by water vapor permeability and the water solubility index.

X-ray diffraction revealed that PAC did not intercalate with the polymer matrix, which indicates that the low interfacial adhesion between the polymer matrix and the filler influenced the properties of the examined composite. The addition of PAC caused a decrease in mechanical strength. On the other hand, the value of Young’s modulus and H_UNHT_ hardness for the tested films changed non-linearly. The highest values were obtained for the films with the highest amount of additive. Furthermore, the conducted measurements showed that small values of roughness characterized the films with low PAC content (1–5%). In contrast, an increase in surface roughness of the tested films was observed in the presence of higher carbon contents. The addition of PAC also influenced the value of the adhesive force. A significant increase of this force was observed for samples with 2–15% of additive. The increase in PAC content caused a rise in water vapor permeability. Measurements of the contact angle showed that the surfaces of films with containing 2% to 5% of additive were hydrophobic, while the base film, C1, C10, and C15 had hydrophilic properties.

The obtained films are biodegradable polymers based on thermoplastic starch derived from renewable natural resources. They are made of a non-toxic material for short utilization and can be used in products such as mulching films, bags, and compostable articles in the agricultural field. This cheaper alternative to conventional packaging, improving food quality and safety, and at the same time reducing costs, can be composted with food waste. In addition, the use of PAC as a filler changes the polymer color, which allows to protect the packaged product from light. The tint modification in packaging materials may also have a marketing function.

## Figures and Tables

**Table 1:** Mechanical properties.

Sample	Thickness [mm]	Maximum Strain ε_max_ [–]	Tensile Strength σ_max_ [MPa]	Young’s Modulus E [MPa]	E_UNHT_ [MPa]	H_UNHT_ [MPa]	ER_NST_ [–]
C0	0.070 ± 0.001 ^a^	0.53 ± 0.08 ^a^	3.40 ± 0.36 ^a^	35.01 ± 3.05 ^abc^	85.25 ± 19.99	5.57 ± 0.70	0.89 ^a^
C1	0.076 ± 0.002 ^b^	0.39 ± 0.04 ^b^	3.09 ± 0.36 ^ad^	37.97 ± 8.68 ^abc^	19.50 ± 4.51	1.64 ± 0.29	0.88 ^a^
C2	0.080 ± 0.002 ^c^	0.31 ± 0.02 ^c^	2.55 ± 0.18 ^bd^	17.95 ± 7.44 ^a^	32.25 ± 5.68	3.08 ± 0.32	0.76 ^a^
C3	0.082 ± 0.001 ^cd^	0.26 ± 0.04 ^ce^	2.43 ± 0.32 ^bc^	24.26 ± 11.64 ^ab^	30.25 ± 5.32	3.73 ± 1.29	0.83 ^a^
C4	0.084 ± 0.001 ^d^	0.28 ± 0.03 ^ce^	3.09 ± 0.12 ^ad^	45.72 ± 3.02 ^bc^	145.00 ± 18.55	8.14 ± 0.18	0.80 ^a^
C5	0.094 ± 0.001 ^e^	0.22 ± 0.02 ^de^	1.86 ± 0.31 ^c^	20.66 ± 8.68 ^a^	90.75 ± 34.53	7.03 ± 1.34	0.81 ^a^
C10	0.095 ± 0.002 ^e^	0.21 ± 0.01 ^de^	3.42 ± 0.27 ^a^	79.65 ± 27.11 ^d^	96.00 ± 17.93	8.49 ± 0.56	0.55 ^b^
C15	0.111 ± 0.003 ^f^	0.16 ± 0.01 ^d^	2.52 ± 0.47 ^bc^	55.08 ± 12.77 ^c^	232.00 ± 71.34	16.51 ± 7.22	0.81 ^a^

^a–f^ Superscripts in the same column mean statistically significantly different groups—Tukey’s HSD test (*p* < 0.05).

**Table 2:** Surface and barrier properties.

Sample	RMS_UNHT_ [µm]	Ra_UNHT_ [µm]	DMT Modulus [MPa]	Adhesion Force [nN]	Water Contact Angle [°]	WSI [%]	WVP [10^−10^ g/(m·s·Pa)]
C0	2.02 ± 0.15	1.64 ± 0.18	685.68 ± 31,40 ^ab^	4.05 ± 0.33 ^ab^	70.11 ± 3.84 ^a^	31.20 ± 0.16 ^ab^	2.60
C1	2.12 ± 0.23	1.71 ± 0.18	637.15 ± 40.24 ^ab^	3,30 ± 1.71 ^a^	86.68 ± 6.67 ^b^	31.45 ± 0.05 ^ab^	3.14
C2	2.19 ± 0.59	1.76 ± 0.53	270.56 ± 143.49 ^a^	10.75 ± 1.02 ^e^	100.64 ± 4.9 ^c^	31.57 ± 0.38 ^ab^	3.27
C3	2.48 ± 0.57	1.86 ± 0.38	514.44 ± 9.16 ^ab^	13.70 ± 1.54 ^e^	101.70 ± 4,24 ^c^	31.44 ± 0.12 ^ab^	3.34
C4	2.20 ± 0.24	1.77 ± 0.18	1052.41 ± 52.29 ^bc^	7.51 ± 0.32 ^bcd^	100.12 ± 2.20 ^c^	32.14 ± 0.26 ^b^	3.34
C5	2.58 ± 0.50	2.07 ± 0.43	337.53 ± 18,77 ^a^	10.72 ± 0.87 ^ce^	102.04 ± 2.87 ^c^	31.95 ± 0.52 ^b^	3.57
C10	3.64 ± 0.98	2.75 ± 0.51	1399,73 ± 491.40 ^c^	8.79 ± 1.92 ^cd^	87.06 ± 5.37 ^b^	30.54 ± 0.55 ^a^	3.96
C15	5.44 ± 01.51	4.47 ± 1.25	772.43 ± 190.1 ^ab^	6.79 ± 1.83 ^abd^	64.82 ± 11.87 ^a^	30.56 ± 0.62 ^a^	4.13

^a–e^ Superscripts in the same column mean statistically significantly different groups—Tukey’s HSD test (*p* < 0.05).

## Data Availability

The data that support the findings of this study are available from the corresponding authors upon reasonable request.
